# Development and testing of zinc sulfate and zinc oxide nanoparticle-coated urea fertilizer to improve N and Zn use efficiency

**DOI:** 10.3389/fpls.2022.1058219

**Published:** 2023-01-17

**Authors:** Bilal Beig, Muhammad Bilal Khan Niazi, Zaib Jahan, Ghulam Haider, Munir Zia, Ghulam Abbas Shah, Zahid Iqbal, Asim Hayat

**Affiliations:** ^1^ Department of Chemical Engineering, School of Chemical and Materials Engineering, National University of Sciences and Technology, Islamabad, Pakistan; ^2^ Department of Plant Biotechnology, Atta-Ur-Rahman School of Applied Biosciences, National University of Sciences and Technology, Islamabad, Pakistan; ^3^ Research and Development Department, Fauji Fertilizer Company Limited, Rawalpindi, Pakistan; ^4^ Department of Agronomy, Pir Mehr Ali Shah Arid Agriculture University Rawalpindi-Arid Agriculture University, Rawalpindi, Punjab, Pakistan; ^5^ Institute of Soil and Environmental Sciences, Pir Mehr Ali Shah Arid Agriculture University Rawalpindi-Arid Agriculture University, Rawalpindi, Punjab, Pakistan; ^6^ Land Resources Research Institute (LRRI), National Agricultural Research Center, Islamabad, Pakistan

**Keywords:** zinc micronutrient, slow-release fertilizer, nanoparticles, zinc sulfate, total organic carbon

## Abstract

Nitrogen (N) losses from conventional fertilizers in agricultural systems are very high, which can lead to serious environmental pollution with economic loss. In this study, innovative slow-release fertilizers were prepared using zinc (Zn) [nanoparticles (NPs) or in bulk], using molasses as an environmentally friendly coating. Several treatments were prepared using Zn in different concentrations (i.e., 0.25%, 0.5%, and 4% elemental Zn). The zinc oxide nanoparticles (ZnO-NPs) were prepared from zinc sulfate heptahydrate (ZnSO_4_·7H_2_O), and were characterized using scanning electron microscopy (SEM), X-ray diffraction (XRD), and Fourier transform infrared (FTIR) spectroscopy. Furthermore, the Zn-loaded urea samples were tested for urea N release rate, leaching of water from soil, and crushing strength to assess the impact of coating on the final finished product. Pot experiments were conducted simultaneously to check the agronomic effects of Zn-coated slow-release urea on the growth and development of wheat (*Triticum aestivum* L.). The laboratory and pot results confirmed that the ZnO-NP treatments boost wheat growth and yield as a result of reduced N and Zn release. UZnNPs2 (urea coated with 0.5% ZnO-NPs and 5% molasses) demonstrated the best results among all the treatments in terms of slow nutrient release, N and Zn uptake, and grain yield. The UZnNPs2 treatment increased plant yield by 34% (i.e., 4,515 vs. 3,345 kg ha^–1^) relative to the uncoated prill-treated crop because of the slower release of Zn and N.

## 1 Introduction

Globally, zinc (Zn) scarcity in soils and plants is a major challenge. Zn is an essential plant micronutrient, which enhances crop yield and quality by contributing to enzymatic reactions (Alloway, 2009; Sheoran et al., 2021). Zn deficiency is present in many of the agricultural lands around the world, but it predominates in temperate and tropical soils of Turkey, Australia, China, India, and Pakistan (Fageria et al., 2003; Cakmak, 2008). Zn deficiency is particularly prevalent in the areas where the soils are saturated with lime (Genc et al., 2006). The calcareous, high phosphorus, and high organic matter content of soils reduces the amount of Zn available to crops (Brennan and Bolland, 2006; Lakshmi et al., 2021). Therefore, the grain produced is Zn deficient, which in turn causes Zn deficiency in animals and humans consuming it. The soil geochemical-related issues reduce the availability of Zn to crops, which in turn affects the quality of food supplies globally (De Almeida et al., 2020). Therefore, it is necessary to recognize the areas of Zn deficiency as well as the key factors that contribute to low supply of Zn to crops. Increasing the Zn content in fields not only enhances the crop yield, but also improves grain quality, which in turn improves human health (Welch and Graham, 2004). Zn is a key element for developing RNA and DNA in both plants and animals. In addition, Zn plays a pivotal role in carbohydrate metabolic reactions and in the structural parts of several proteins (Prasad, 2008; Maret, 2009). Farmers in underdeveloped countries mostly apply nitrogenous fertilizers directly to crops without the addition of sufficient levels of phosphorus and other micronutrients. In addition, more than one crop cycle within the same year might also result in high levels of Zn deficiency within soil systems (Yaseen and Hussain, 2021).

Zinc sulfate (ZnSO_4_) bulk salt is commonly used as a source of Zn in calcareous soils, and it is highly soluble. Conversely, zinc sulfate can rapidly convert to an insoluble form after complex reactions with soil components. In the long term, this insoluble form turns out to be useless for the plant–soil system, especially in calcareous soils (Hussain et al., 2012; Yaseen and Hussain, 2021). Zinc oxide (ZnO) is also used as a Zn source because of its low cost; however, it is not commonly applied to fields, as it is less soluble in calcareous soils (Hussain et al., 2012). Rather, the fertilizer (ZnO) is preferred to be used in acidic soils. There is a complex mechanism involved within soil behind the addition of Zn and its availability to plants. The availability of Zn highly depends on the fate of its parent mineral in the soil and its interaction with soil constituents. The pH and moisture content of the soil, and the atmospheric conditions, also affect Zn availability. The addition of very small amounts of Zn (i.e., 2%–5%) to fields is usable by plants, while the remaining Zn gets fixed by the soil, although its residual effect has been reported over subsequent years (Rashid et al., 2017). To increase Zn use efficiency in plant–soil systems, many schemes have been adopted that use different Zn salts with various application methods (soil or foliar, or a mixture of both) (Montalvo et al., 2016). Other techniques are in use as well as inorganic fertilization. These techniques also utilize and promote natural and biological processes such as soil acidification, chelation, interaction with phosphorus, and preferential release using microorganisms (Subramanian et al., 2008).

Nanofertilizers that are being used now are getting much attention in the field of agriculture because of their high solubility, availability, diffusion, and reactivity in the soil (El-Saadony et al., 2021). These properties are attributed to having a smaller size, higher surface area, and higher surface energy, as compared with their bulk salts. As a result, zinc oxide nanoparticles (ZnO-NPs) can be applied as a fertilizer for better growth of crops and for minimizing the Zn deficiency in crops. The effects of ZnO-NPs are also dependent upon the dosage, rhizosphere atmospheric conditions, nature of the soil, and moisture content (Mukherjee et al., 2016; García-Gómez et al., 2017; Dimkpa et al., 2019b). However, the distinction between toxic and beneficial impact of ZnO-NPs is mainly limited to its application rate to plants. Therefore, the precise proportion of ZnO-NPs to be used must be calculated and applied to crops by evaluating the whole ecosystem (Elmer et al., 2018; Adisa et al., 2019; Dimkpa et al., 2019a; Dimkpa et al., 2019b).

Nanofertilizers have great benefits, including higher nutrient use efficiency and plant yield at lower application rates, without affecting the productivity (Kottegoda et al., 2017). Therefore, using nanofertilizers could decrease the cost of fertilizing plants and pollution, linked with soil and water leaching, by making a sustainable and cleaner product (Dos Santos et al., 2015). The commercial usage and adaptation of ZnO-NPs is a bit slower because of the possible environmental and health problems associated with it. The major issue in the use of dry nanoparticles is their dust, which can cause serious health concerns on inhalation (Bakshi et al., 2015; Fatima et al., 2021). Furthermore, excessive applications under moist soil conditions might lead to their leaching into the underground water sources, which can in turn affect the body of water and life associated with it (Krysanov et al., 2010). The activity and effectiveness can be reduced in aqueous media by conversion of the nanoparticles into their ionic form or into their micro-scale salts (García-Gómez et al., 2017; Qiu and Smolders, 2017). However, the aggregation and agglomeration of nanoparticles leads toward micro-scale particles, which affects the size-specific reactivity of the nanoparticles (Beig et al., 2022).

The combination of most common N fertilizer (urea) particles with ZnSO_4_ causes particle size-dependent segregation when blending or packaging is done. The problem of particle separation escalates when ZnO-NPs are blended with prills. Therefore, it is important to improve the nutrient delivery system to crops without any loss in efficiency and combine the use with conventional urea particles. The method would, therefore, help to supply multiple nutrients within a single fertilizer dose. The techniques used for supplying multiple nutrients with finished products of urea, NPK (nitrogen : phosphorus : potassium), or diammonium phosphate (DAP) include coating and encapsulation of nanoparticles (Kottegoda et al., 2017; Beig et al., 2020b; Beig et al., 2020c; Dimkpa et al., 2020b). A number of research studies have been performed to investigate the effect of ZnO-NPs coatings on urea granules (Dimkpa et al., 2019b; Dimkpa et al., 2020a; Dimkpa et al., 2022). In addition, Zn dissolution kinetics from urea was only reported in a few research studies conducted by Milani et al. (Milani et al., 2012; Milani et al., 2015). By using nanomaterials, environmentally friendly and cleaner nanofertilizers can be synthesized, which in turn can reduce the nutrients to be applied and the nanofertilizers can be applied at a lower rate to fields (Derosa et al., 2010; Dimkpa et al., 2020b; Das and Ghosh, 2021).

The novelty in the current study is to prepare an innovative, slow-release urea N fertilizer using Zn that could perform dual functions, i.e., act as a slow-release coating and as a micronutrient. The major objectives of the research were to (i) synthesis and characterize ZnO-NPs prepared using ZnSO_4_ as a precursor and urea coated with ZnO-NPs, (ii) assess different percentages of ZnO-NPs and ZnSO_4_ bulk salt with molasses coatings on fertilizer properties and growth-related parameters of the wheat crop, and (iii) evaluate the impact of a lower dose of ZnO-NP-coated urea in comparison to its bulk counterpart coated urea at a higher dose. Finally, all the effects of bulk-coated urea and ZnO-NP-coated urea were compared and analyzed for the determination of an optimum dose of nanoparticles.

## 2 Materials and methods

### 2.1 Reagents

Chemicals such as zinc sulfate heptahydrate, sodium hydroxide, and deionized water were obtained from Sigma-Aldrich Chemical Company. Sugar cane molasses (86° Brix) and a commercial prilled urea bag containing 46% N was purchased from a local market. For the removal of dust and broken particles of urea, sieving was done prior to use in the fluidized bed coater.

### 2.2 Synthesis of ZnO-NPs using ZnSO_4_

The Zn nanoparticles were synthesized using a wet precipitation method. The procedure started with the preparation of solutions of 500 mL of 1 M ZnSO_4_·7H_2_O and 500 mL of 2 M NaOH in double-distilled water. The 1 M ZnSO_4_ solution was positioned on a hot plate, with a stirrer, and, after some time, 2 M NaOH solution was added, dropwise, to maintain the solution pH. The color of the ZnSO_4_ solution started to turn white with the formation of gels after the addition of NaOH. The solution needed continuous stirring for the formation of the white suspension. This suspension was left overnight, with continuous stirring. The white suspended materials were filtered out using filter paper. The precipitates were thoroughly washed with distilled water to remove excess NaOH. These precipitates, after washing, were placed in an oven at 80°C for 12 h. After the drying process, the material was grounded into fine powder to obtain the nanoparticles (Tarafder et al., 2020).


$$ZnSO_{4}\cdot 7H_{2}O+2NaOH+H_{2}O\to Zn{\left(OH\right)}_{2}+Na_{2}SO_{4}+8H_{2}O$$

$$Zn\;{\left(OH\right)}_{2}\to \;ZnO+H_{2\;}O$$

### 2.3 Solution synthesis and coating process

The coating process was started by preparing a homogeneous solution of ZnO-NPs in deionized water. Molasses was also added. The solution was then sonicated for 1 h in a sonication bath. The same methodology was also adopted for the bulk ZnSO_4_ salt solution. The composition of the coating formulation is detailed in the **Table 1**. The coating process of the urea prills was performed using fluidized bed coater YC-1000 (Pilotech, Shanghai, China). The sieved urea prills were loaded in the fluidized bed chamber. The process parameters of coating were adopted, with slight changes, following the previous study by Beig et al. (Beig et al., 2020b). A total of 1,000 g of prilled urea was loaded into the chamber. The chamber temperature was maintained at 90°C using hot dry air. The hot coating solution (80°C) was showered using a bottom-mounted spray nozzle. The coating process was completed with drying step using hot air at 100°C for 5 min.

**Table 1:** Composition of the coating formulations.

Coating Formulation	Weight %/100 g of urea		
	ZnSO_4_	ZnO-NPs	Molasses
UC	–	–	–
UZnS1	0.25	–	5
UZnS2	0.5	–	5
UZnS3	–	–	5
UZnNPs1	–	0.25	5
UZnNPs2	–	0.5	5
UZnNPs3	–	4	5

### 2.4 Characterization of ZnO-NP- and ZnSO_4_ bulk salt-coated urea

The synthesized nanoparticles were tested using X-ray diffraction (XRD), Fourier transform infrared spectroscopy (FTIR), and scanning electron microscopy (SEM). The uncoated and coated urea prills were tested with UV–VIS spectroscopy for determining the release of urea N. The Zn discharge rate from the prepared formulations was calculated from a leaching experiment with the help of an atomic absorption spectroscopy technique. A percolation reactor was utilized for this test.

#### 2.4.1 Physical and structural characterization

X-ray diffraction analysis of the synthesized nanoparticles was performed using equipment from STOE Germany. The step size was kept at 0.4 s^–1^. The scan angle was maintained from 20° to 80°, with a voltage of 40 kV (Cakmak et al., 2010). FTIR was performed to check the different functional groups in the ZnO-NPs. This technique was performed on PerkinElmer Spectrum 100 FTIR spectrometer at a wave number range of 4,000–400 cm^–1^. The ZnO-NPs were suspended in double-distilled water using a mechanical stirrer and ultraprobe sonication machine (100 W, 40 kHz, and 30 min). The nanoparticles needed vigorous stirring to avoid the agglomeration of particles for morphological examination (Adhikari et al., 2015). SEM (S-4700, Hitachi, Japan) was used for this purpose. Gold sputtering was performed before analysis (Beig et al., 2020c).

#### 2.4.2 Urea N release rate

The release rate of urea N and the efficiency of the coated samples were determined by following the *p*-methyl amino benzaldehyde method. To quantify the nutrient release from the prepared samples, the following method was used.

##### 2.4.2.1 *p*-Methyl amino benzaldehyde method

A total of 10 g of coated prills was placed in a 5-L beaker, which was filled with deionized water. The next step was the collection of a 10-mL sample from the beaker at a range of time intervals: 3, 6, 9, 12, 15, 30, 60, and 120 min. After sample collection, dilution was done, followed by the addition of 1 mL of acid solution and 5 mL of coloring agent. The final step involved absorbance measurement at 418 nm. The urea concentration at different intervals was calculated using Eq. (1). The efficiency of different formulations was calculated using Eq. (2) (Al-Zahrani, 2000):


$$Urea\;\left(ppm\right)=\;absorbance-Y.\frac{intercept}{slope}of\;calibration.\;\left(1\right)$$

$$Efficiency\;\left(\%\right)=Cu-\frac{Ccu}{Cu}*\;100,\;\left(2\right)$$

where, *C_u_* and *C_cu_* are the urea concentration (ppm) in the uncoated and coated prills, respectively, at 15 min.

#### 2.4.3 Zn Leaching using sand column

A percolation reactor was utilized to determine the level of leaching of Zn from the Zn-loaded urea. The apparatus was similar to the one used by Hernandez et al. (Hernández et al., 1994). A constant flow (20 mL) of double-distilled water was maintained through the sand column. For the experiments, within the column, two layers of experimental soil with 50 (50-g) zinc-treated urea samples were placed between the soil layers. In the percolation reactor, 15–20 g of experimental soil was used. The solution was collected after different time intervals (24, 48, and 72 h) for testing of the Zn concentrations in the water. The Zn contents were analyzed with atomic absorption spectroscopy. The whole experiment was carried out at 25°C (Yuvaraj and Subramanian, 2015; Yuvaraj and Subramanian, 2018).

### 2.5 Pot experimentation

A standard pot test was conducted using soil collected from the ARID Agricultural University research farms in Rawalpindi, Pakistan. The collected soil was first sieved through a 2-mm screen to remove debris composed of roots, shoots, and leaves. After screening, 16 kg of soil was placed into each pot. The diameter and depth of each pot were 26 and 24 cm, respectively. Overall, eight treatments were used for this study. The treatments used in the present study were: control (C), treated with no Zn-loaded fertilizer; UC, uncoated urea prills; UZnNPs1, urea prills coated with 0.25% elemental zinc (as ZnO-NPs) and 5% molasses; UZnNPs2, urea prills coated with 0.5% elemental zinc (as ZnO-NPs) and 5% molasses; UZnNPs3, urea prills coated with 4% elemental zinc (as ZnO-NPs) and 5% molasses; UZnS1, urea coated with 0.25% elemental zinc (as ZnSO_4)_ and 5% molasses; UZnS2, urea coated with 0.5% elemental zinc as ZnSO_4_ and 5% molasses; and UZnS3 urea coated with 4% elemental zinc (as ZnSO_4_) and 5% molasses. Recommended amounts of potassium- and phosphorus-based fertilizers (30 kg ha^–1^ and 87 kg ha^–1^, respectively) were also applied to all the pots, including the control treatment. All the pots were positioned in a completely randomized design (CRD) in an open field, with three replicates of each treatment. The wheat (*Triticum aestivum* L.) seeds were sown manually. After germination, six healthy plants were maintained per pot. All the treatments were applied at the time of seed germination. The wheat was harvested, at its physiological maturity, after 5 months from sowing. The formulated prills were placed on the top layer of the soil (Aziz et al., 2019).

### 2.6 Soil biochemical analysis

To check the impact of Zn-coated fertilizer on soil properties, samples were taken at four different intervals throughout the pot experiment. The first soil sample was collected before fertilizer application. The remaining soil samples were collected as the pot experiment proceeded, i.e., 40, 86, and 115 days after sowing of the wheat. The last soil sample was collected before the final harvest of the wheat (i.e., at 140 days). Every sample of soil was tested to check its total organic carbon (TOC), dissolved organic carbon (DOC), mineral N, and microbial biomass C, N, and Zn content. All the tests were performed using the methodology reported by the International Center for Agricultural Research in the Dry Areas (ICARDA) soil and plant analysis methods manual (Munir et al., 2020; Sahu et al., 2022). The experimental properties of soil are given in **Table 2**.

**Table 2:** Properties of the experimental soil used for the pot tests.

Soil property	Unit of measurement	Value
pH	–	7.85
Electrical conductivity	dS m^–1^	0.85
Organic matter content	%	0.41
TOC	%	0.30
Available C	%	0.14
Mineral N	ppm	1.79
Extractable potassium	ppm	154
Olsen phosphorus	ppm	2.84
DTPA–Zn	ppm	0.17

DTPA, diethylenetriaminepentaacetic acid.

### 2.7 Chemical analysis of experimentation soil

The pH and electrical conductivity were measured using a soil and water suspension in the ratio of 1 : 2.5, which was allowed to settle overnight at room temperature to achieve equilibrium conditions (Page et al., 1982). The soils were treated with chromic acid, hydrogen peroxide, and sulfuric acid to calculate the carbon content (Walkley and Black, 1934). The Kjeldahl digestion process was used for calculating plant and soil total N content. The plant’s available fraction of soil zinc was determined using the diethylenetriaminepentaacetic acid (DTPA) method (Lindsay and Norvell, 1978).

#### 2.7.1 Soil microbial biomass C and N

The C and N contents of the soil microbial biomass [i.e., soil microbial biomass (MBC) and soil microbial biomass N (MBN)] were assessed with the fumigation–extraction procedure (Brookes et al., 1985; Vance et al., 1987). The test procedure started with the collection of a 10-g sample of soil. These samples were further separated into two identical portions. Ethanol-free chloroform was utilized for fumigation of 5 g of the sample at room temperature for 1 day. Then extraction was done with potassium sulfate, followed by shaking the samples in a reciprocal shaker for 30 min. A similar methodology was adopted for non-fumigated soil. A TOC analyzer was used for the determination of total organic carbon. The N content was determined using the Kjeldahl digestion method.

MBC and MBN values were computed using Eq. (3).


$$MBC\;or\;MBN=\;TC\;or\;TN\;–TC\;or\frac{TNnf}{kEC}or\;KEN,\;\left(3\right)$$

where TNf and TNnf are total N in fumigated and non-fumigated soil, respectively. kEC and kEN are 0.45 and 0.54 and are utilized during the calculation of MBC (Jenkinson et al., 2004) and MBN (Brookes et al., 1985).

#### 2.7.2 Extraction of Zn from microbial biomass

The Zn present in the soil microbial biomass was extracted using the lysis method in the presence of chloroform. A similar method was followed as was utilized for fumigation extraction. However, 2 mL of 1 M ammonium nitrate was used instead of potassium sulfate. The reaction mixture was filtered and then the extracted solution was acidified with nitric acid. The Zn content was determined with an atomic absorption spectrophotometer. The concentration of Zn was then determined by subtracting the Zn content in a non-fumigated soil from that in a fumigated sample (Aziz et al., 2019).

### 2.8 Wheat analysis

The plant samples for all the treatments were harvested at physiological maturity. The grain yield of each treatment was recorded, and the parts of wheat plants were analyzed for mineral contents. The harvested plant shoot and fresh biomass were measured immediately after harvest. All the wheat plants were initially dried at 70°C for 2 days to estimate the dry matter content of the wheat crop. The wheat plant roots were removed from pots followed by washing in water. After removal of the soil, the plant’s fresh weight was calculated. The roots were then oven dried for 48 h to determine the root dry matter content. Different parts of the plant were also tested for Zn content. The chlorophyll content of wheat plants was determined just before the harvesting with the help of a SPAD chlorophyll meter (Sadaf et al., 2017).

#### 2.8.1 Plant N and Zn uptake

The N content of wheat tissues for all the formulations was determined with the help of the Kjeldahl digestion process. One gram of dried biomass of wheat plant was used for the digestion. The plant tissue was placed in 5 mL of concentrated sulfuric acid in digestion tubes. The reaction temperature was slowly increased until it reached 145°C. The tissue was then incubated at this temperature for 1 h, following which 5 mL of tri-acid mixture was added. The mixture temperature was increased further to 240°C for the next 1 h. Then, all the samples were allowed to cool down. The samples were then filtered. The final filtrate was used in an atomic absorption spectrophotometer for elemental Zn detection (Aziz et al., 2019).

### 2.9 Statistical analysis

The statistical analysis of all the experimental results was performed using SPSS statistics v. 19.0 (IBM, New York, NY, USA). Analysis of variance (ANOVA) was used to study the effect of formulated fertilizer. The test results were presented as the means and standard errors (SEs) and were calculated from three replications (mean ± SE; *n* = 3). The significance of differences between various treatments was explored at a 5% probability level. The least significant difference (LSD) test was performed to evaluate the multiple differences, with a *p-*value of an ANOVA of ≤ 0.05 being considered significant.

## 3 Results

### 3.1 Morphological, structural, and chemical characterization of ZnO-NPs

**Figure 1:**
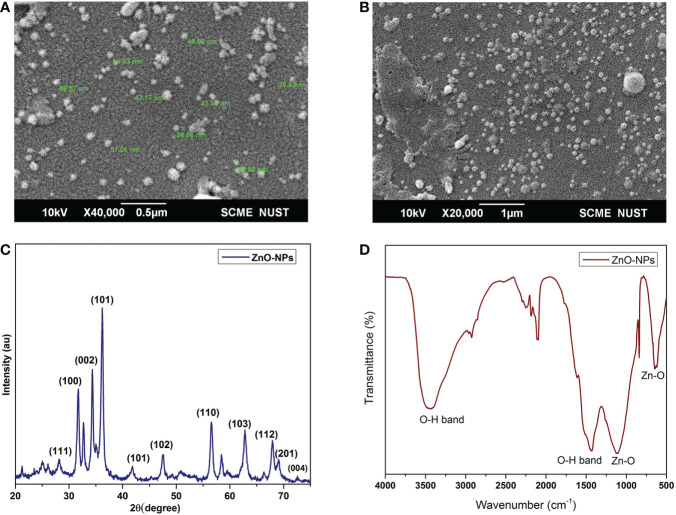
Scanning electron microscope images of ZnO-NPs. **(A)** ×40,000 magnification; **(B)** ×20,000 magnification; **(C)** XRD spectra of ZnO-NPs; and **(D)** FTIR of ZnO-NPs.

Scanning electron micrographs of the ZnO-NPs are shown in **Figures 1A, B**. The nanoparticles depicted in **Figures 1A, B** are circular and slightly spherical in shape. Particles with dimensions less than 50 nm are clearly seen in the micrographs. The ZnO-NPs were well separated from each other. A very small agglomeration was seen in the SEM micrograph. XRD results clearly displayed the peaks of ZnO-NPs, which were very prominent, as shown in **Figure 1C**. The ZnO-NPs peaks at 2Ѳ = 31.9, 34.45, 36.35, 47.6, 56.65, 62.9, 66.4, 67.9, 69.1, 72.6, and 76.9 were seen in the X-ray diffractogram. The infrared spectrum of nanoparticles is shown in **Figure 1D**. Analysis was performed using the KBr method. The spectrum of ZnO-NPs showed bands for different functional groups at 3,343, 1435, 1110, 645, and 545 cm^–1^, as shown in **Figure 1D**.

### 3.2 Characterization and testing of coated prills

#### 3.2.1 Release rate of urea N

**Figure 2:**
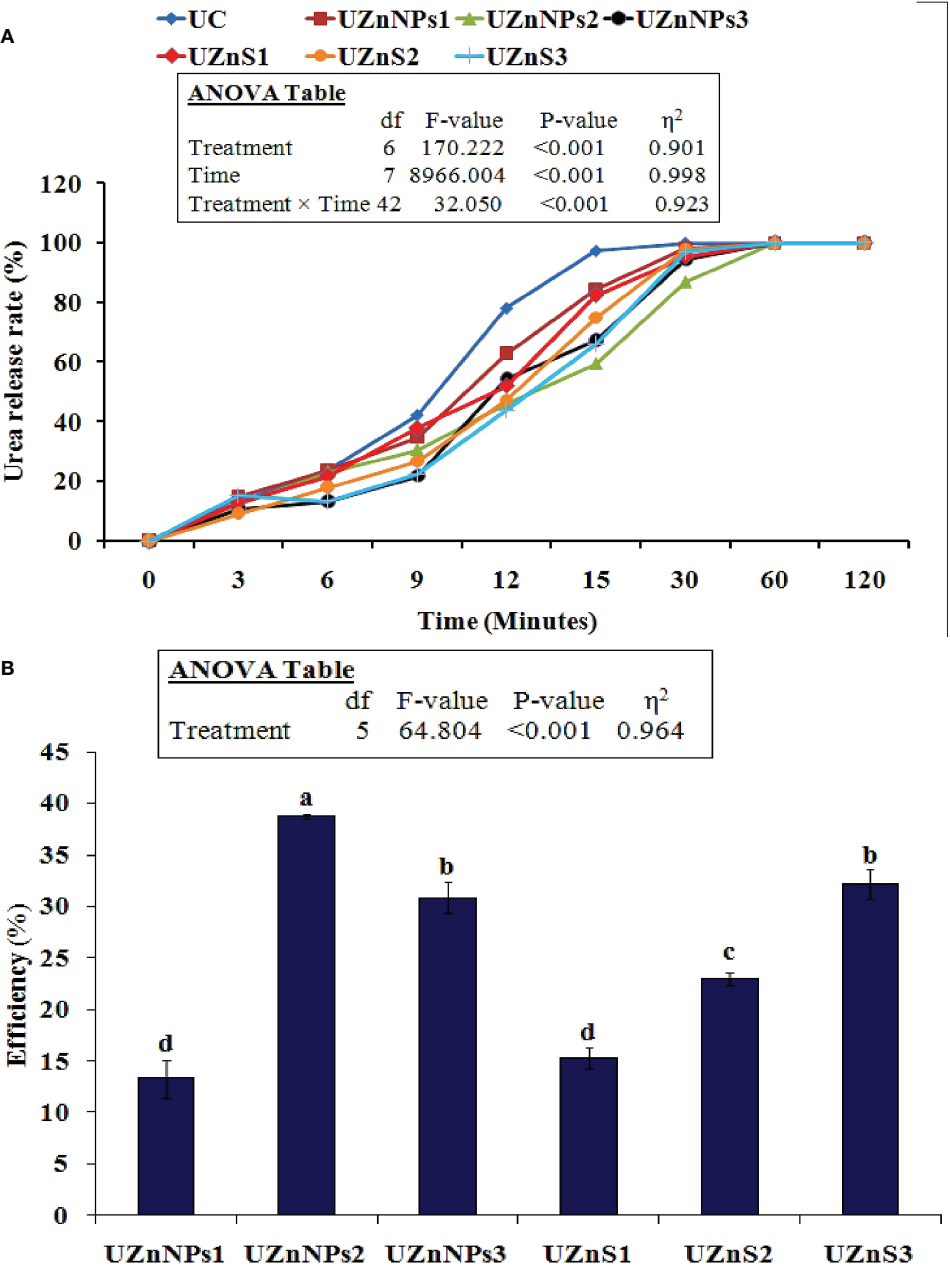
**(A)** Impact of the coating on urea release rate for different formulations. **(B)** Coated urea efficiency at 15 min.

The time release profile of all seven treatments is summarized in **Figure 2A**. The total urea N released from the coated and uncoated urea was calculated as a function of time at room temperature using Eq. 1. In all the treatments, the urea concentration gradually increased and then dropped over time. The initial release of uncoated urea N was higher than in the coated samples. On the other hand, the Zn coatings slowed down the N release, depending on the amount of coating material used. The treatment UZnNPs2 showed the slowest release of urea. The urea N release from the uncoated urea generally occurs very abruptly. This process takes place quickly, which mimics the actual behavior of urea when it comes in contact with water, because of its higher solubility. The Zn and molasses coating reduced the N release from prilled product, which meets the needs of the plant. In summary, the coating materials are first dissolved after coming in contact with water, and this then allows the water to get in contact with the fertilizer core. All the coatings significantly slowed the urea release, as shown in **Figure 2A**. After 30 min, in all the coated treatments, the remaining urea N was released in a catastrophic manner, just like uncoated urea. The results presented in **Figure 2B** demonstrate the efficiency of coated urea at 15 min. The highest efficiency was seen for UZnNPs2 formulation, i.e., 39%, whereas the UZnNPs1 exhibited the lowest efficiency among all the coated treatments, i.e., 13%.

#### 3.2.2 Water–soil Zn leaching

**Figure 3:**
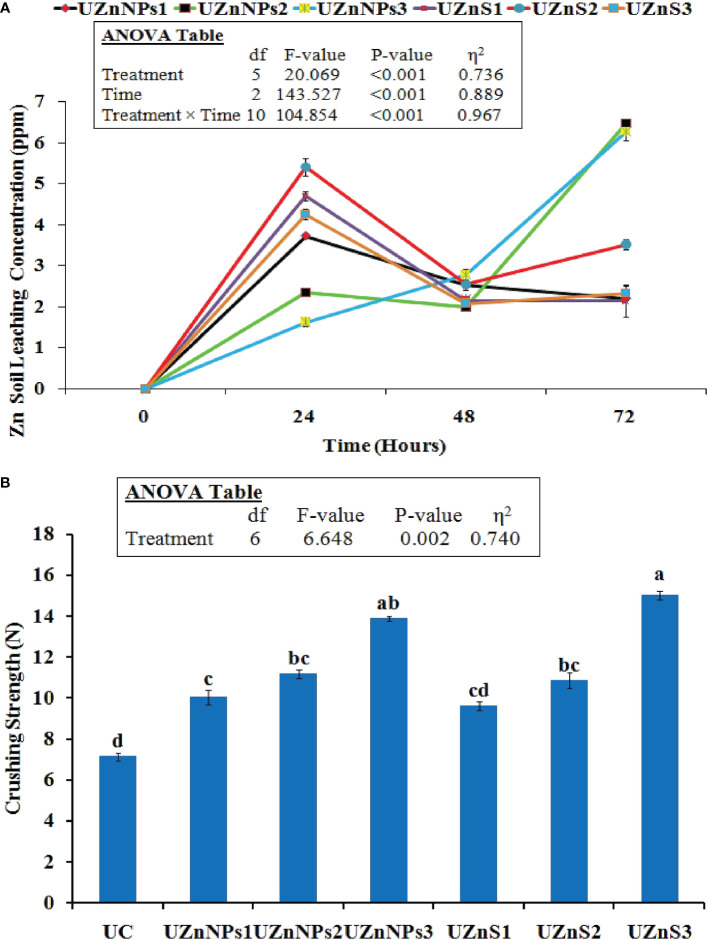
**(A)** Zn concentration in leachate from the sand column experiment. **(B)** Impact of coating on the crushing strength of prills. Control (C), treated with no fertilizer applied; UC, uncoated urea prills; UZnNPs1, urea prills coated with 0.25% elemental zinc (as ZnO-NPs) and 5% molasses; UZnNPs2, urea prills coated with 0.5% elemental zinc (as ZnO-NPs) and 5% molasses; UZnNPs3, urea prills coated with 4% elemental zinc (as ZnO-NPs) and 5% molasses; UZnS1, urea coated with 0.25% elemental zinc (as ZnSO4) and 5% molasses; UZnS2, urea coated with 0.5% elemental zinc (as ZnSO4) and 5% molasses; and UZnS3, urea coated with 4% elemental zinc (as ZnSO4) and 5% molasses.

The Zn concentration pattern of all the prepared formulations in the sand column is was given in **Figure 3A**. The water leachate readings were recorded on a daily basis, for 3 consecutive days. After 24 h, the highest Zn values in leachate were observed in the UZnS2 formulation, i.e., 5.39 ppm. Initially, the Zn release patterns of all the treatments increased over a 24-h period, with a gradual slowing over the next 2 days. However, the case was a bit different for UZnNPs2 and UZnNPs3 treatment formulations, in which Zn concentration increased quickly after 72 h, i.e., 6.45 ppm and 6.27 ppm, respectively. The experimental data of all the formulated treatments revealed that the coated Zn on prills was exhausted 3 days after the start of the experiment. However, the addition of molasses to ZnSO_4_ and ZnO-NPs facilitated the slowing of the release of Zn and urea; this slowing meets the needs of plants.

#### 3.2.3 Crushing strength

The crushing strength of all the urea formulations is displayed in **Figure 3B**. All the coated treatments enhanced the impact strength of the prills. The test values of all the coated fertilizer were significantly higher than those of the uncoated prill (7.15 N), as shown in **Figure 3B**. UZnS3 treatment exhibited the highest crushing strength among all the prepared samples, whereas the 0.25% ZnSO_4_ coating yielded the lowest crushing strength among all the coated formulations. The molasses percentage was constant in all the coating formulations, i.e., 5%. The molasses in the coating performs as an adhesive to bind the nano- or bulk Zn. Therefore, the presence of molasses promoted the strength of the coating layer, which probably enhanced the resistance against external impact. The treatments with the highest percentage of coating material (UZnNPs3 and UZnS3) increased the crushing strength more, as shown in **Figure 3B**.

### 3.3 Biochemical examination of soil

**Figure 4:**
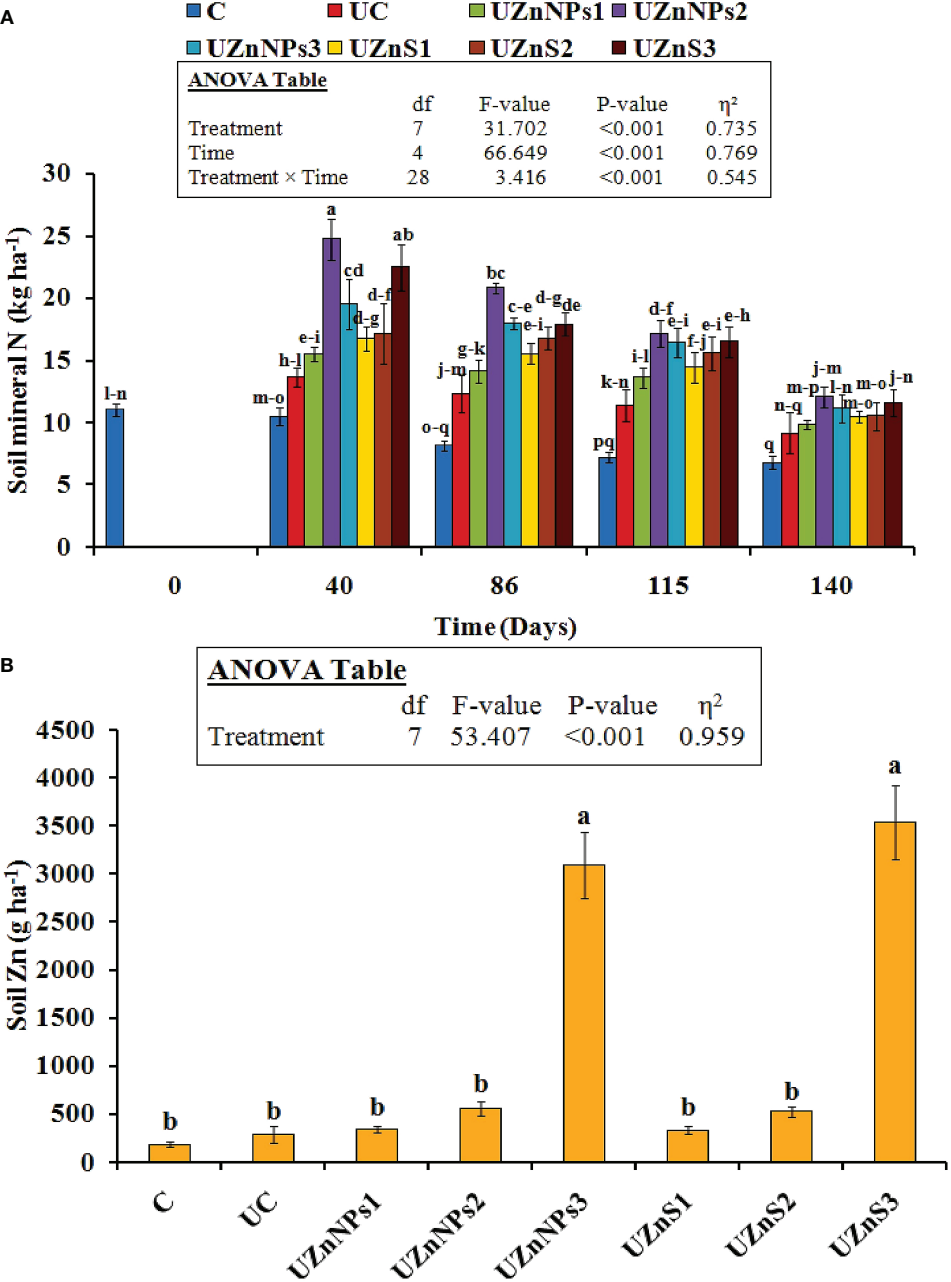
**(A)** Soil mineral N content at different growth stages (i.e., at 40, 86, 115, and 140 days). **(B)** Soil Zn after fertilizer amendment at harvesting stage.

The Nmin present in the soil was also greatly improved by the addition of urea treatments with respect to time (*p*<0.001), as depicted in **Figure 4A**, whereas, the different treatments of Zn-coated urea did not show any significant change among the applied bulk ZnSO_4_ salt and ZnO-NPs. The blending of Zn and molasses for prills coating probably boosted the Nmin values relative to the uncoated and control treatments. The difference in Nmin was clearly significant in the formulations with nano-Zn. In general, the Nmin in all the applied treatments decreased with respect to time. However, in each formulation, this change did not vary considerably at different stages (i.e., 40, 86, 115, and 140 days). The highest value of Nmin was assigned to UZnNPs2, which increased its value twofold (24.7 kg ha^–1^ vs. 10.5 kg ha^–1^), compared with the control treatment, on the 40th day of the experiment. The smallest increments in Nmin values were observed at the lowest rate of ZnO-NPs among all the Zn-coated treatments (i.e., 0.25% elemental Zn). The application of Zn-coated prills greatly enhanced the soil DTPA-Zn, as presented in **Figure 4B**. But their effect was more prominent in the treatments with the highest percentage of elemental Zn, irrespective of the source of Zn (i.e., ZnSO_4_ or ZnO-NPs). The highest values of soil DTPA-Zn were seen in the UZnS3 and UZnNPs3 treatments, i.e., 3,541 g ha^–1^ and 3,095 g ha^–1^, respectively. The lowest value was observed for the UZnS1 treatment, i.e., 337 g ha^–1^.

**Figure 5:**
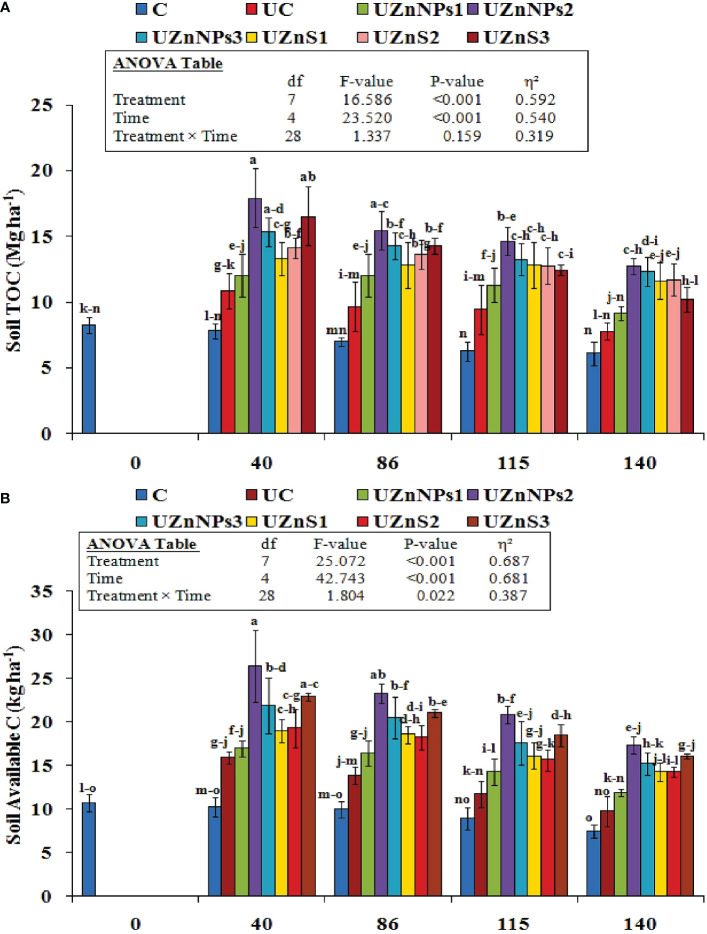
**(A)** Soil total carbon (TOC) and **(B)** available carbon (DOC) in soil at different stages.

The results of soil total organic carbon (TOC) and soil available C for all the treatments are shown in **Figures 5A, B**, respectively. The addition of Zn-loaded urea significantly enhanced the soil TOC and dissolved organic carbon (DOC), as represented in **Figures 5A, B** (*p*<0.001). The soil TOC was greatly influenced by the different zinc urea treatments, time, and soil interaction (*p*<0.001), as indicated in **Figure 5A**. **Figure 5A** shows that soil TOC values were highest after 40, 86, 115, and 140 days, that is, they were, respectively, 64% (17.9 kg ha^–1^ vs. 10.9 kg ha^–1^), 59% (15.5 kg ha^–1^ vs. 9.7 kg ha^–1^), 53% (14.6 kg ha^–1^vs. 9.5), and 64% (12.8 kg ha^–1^vs. 7.8 kg ha^–1^) higher in the soil samples modified with UZnNPs than in UC. Alternatively, the soil amended with the UZnNPs1 fertilizer showed the least impact among all the Zn-coated treatments, which remained constant (12 mg ha^–1^) for 40 and 86 days. The soil TOC values of all the treatments gradually decreased as the experiment proceeded. The soil samples collected after 140 days registered a significant decrease in soil TOC values for all the treatments. A significant increase in the soil DOC was noted with the addition of zinc-treated urea. **Figure 5B** shows that the highest value of soil DOC for UZnNPs2-treated soil (i.e., 26.4 kg ha^–1^) occurred on the 40th day of the experiment. The increased amount of ZnO-NPs and ZnSO_4_ enhanced the DOC values of soil, as depicted in **Figure 5B**. The soil DOC values gradually decreased, but the fertilizer treatments continuously kept on affecting the soil parameters after 140 days. The lowest increase in soil DOC was observed for the pots amended with UZnNPs1 (i.e., 11.9 kg ha^–1^) after 140 days.

### 3.4 Soil microbial biomass C, N, and Zn

**Figure 6:**
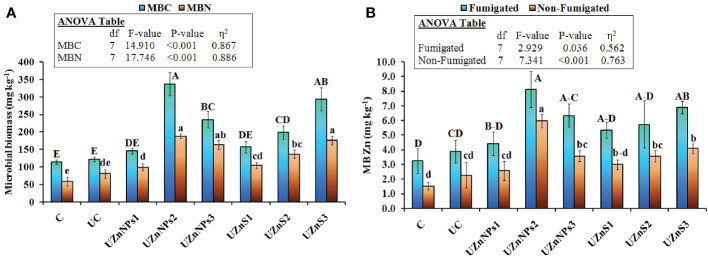
**(A)** Microbial biomass carbon (MBC) and microbial biomass nitrogen (MBN), and **(B)** microbial biomass Zn in fumigated and non-fumigated samples after fertilizer application.

The UZnNPs2 treatment was applied for crop growth, and greatly enhanced the soil MBC, MBN, and MBZn, relative to all other fertilizer treatments, as demonstrated in **Figures 6A, B** (*p*<0.001). Addition of Zn and molasses (co-mixing) greatly enhanced the MBC and MBN compared with the uncoated and control treatments. The increasing percentage of Zn-amended urea prills enhanced the MBC and MBN more, relative to the lowest rate of nano- or bulk Zn. The MBC and MBN values for ZnO-NP-coated urea were significantly higher than their bulk salts. Both the MBC and MBN values were almost similar for the control, uncoated, and the lowest rate of applied Zn (i.e., 0.25% elemental Zn), as shown in **Figure 6A**. Overall, an increasing trend was observed in the MBC and MBN values at higher percentages of Zn amendment to the soil. The Zn content in both fumigated and non-fumigated samples was significantly different from that of the uncoated and control soil samples, as plotted in **Figure 6B** (*p*<0.05). The highest value of Zn in fumigated soil was observed for samples amended with 0.5% elemental Zn. The increasing effect on MBZn was more prominent in pots amended with ZnSO_4_-coated urea. Alternatively, ZnO-NP-amended soil yielded much higher values of Zn, as shown in **Figure 6B**. The MBZn value for UZnNPs2 was 8.13 mg kg^–1^, compared with 3.25 mg kg^–1^ and 3.90 mg kg^–1^ for the control and uncoated treatments, respectively.

### 3.6 N and Zn uptake by wheat

**Figure 7:**
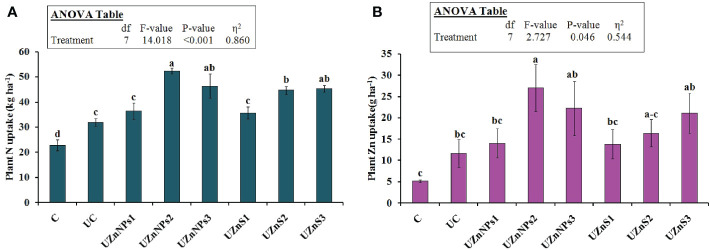
**(A)** Plant N uptake and **(B)** plant Zn uptake after urea prill application.

The crop N uptake was accelerated in all the Zn (nano- or bulk)-coated prills, relative to the treatments with no fertilizer, as depicted in **Figure 7A**. The highest value of N uptake was observed in the treatment containing 0.5% elemental Zn (nano), relative to all other treatments. The effect of nano-Zn-coated treatments was significantly more prominent in plant N uptake relative to the bulk ZnSO_4_-coated urea. Like N uptake, the values of plant Zn uptake were also enhanced by the addition of coated prills in comparison with pots treated with no fertilizer, as shown in **Figure 7B**. Increasing Zn percentage in coatings enhanced Zn uptake in wheat regardless of the Zn source (nano or bulk). The formulations with 0.5% and 4% Zn (nano or bulk) significantly boosted the N and Zn uptake, whereas 0.25% elemental Zn-amended prills gave median values of N and Zn uptake, when compared with uncoated prill-treated soil. The plant N and Zn uptake values revealed ZnO-NP-coated formulations to be more beneficial for enhancing the N and Zn accumulation in wheat than bulk salt ZnSO_4_.

### 3.7 Agronomic parameters of wheat

**Figure 8:**
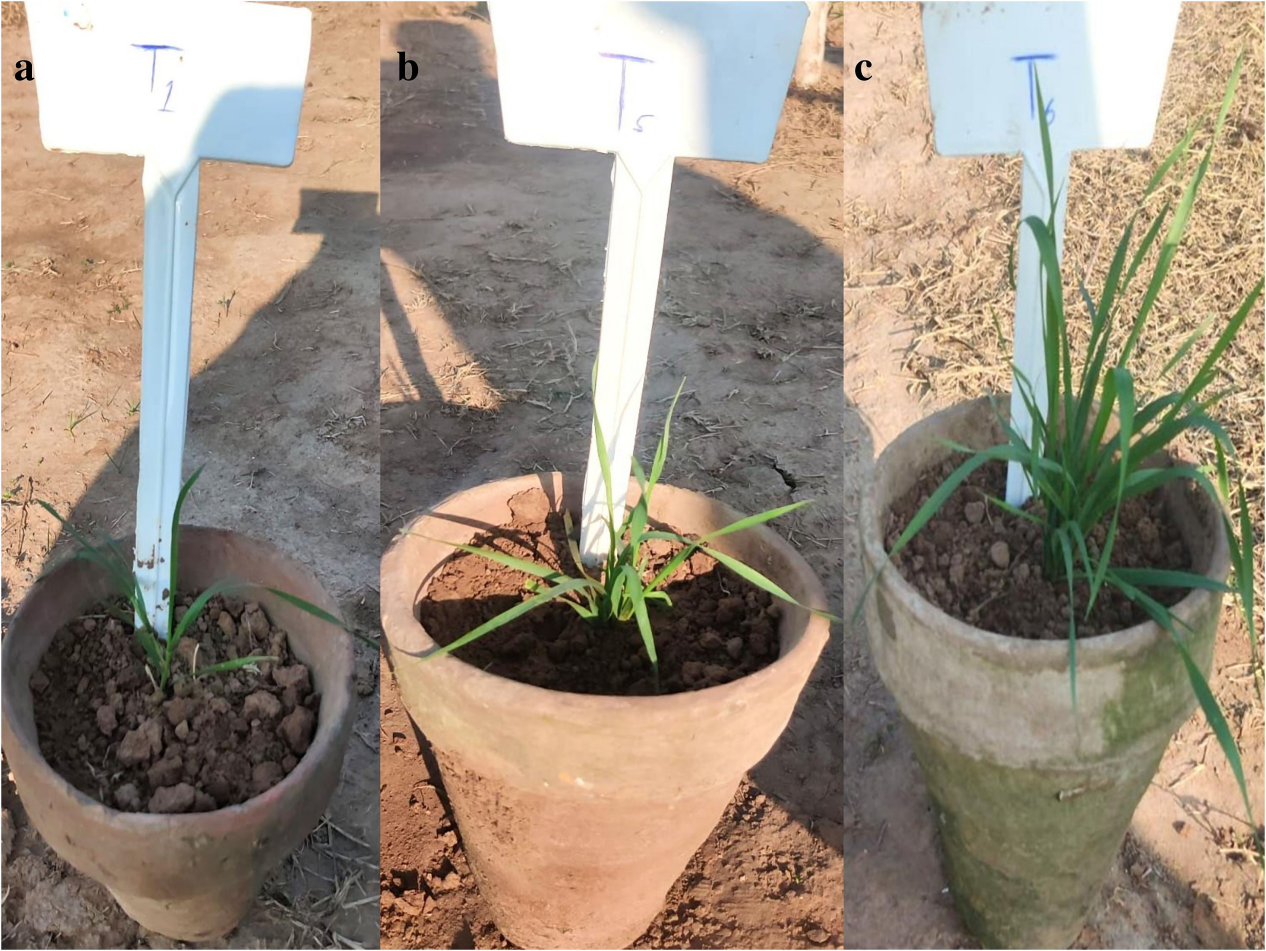
Pictorial representation of the pot experiment, **(A)** Control; **(B)** UZnS2 treated; and **(C)** UZnNPs treated.

The wheat yield and physiological attributes were greatly influenced by the coating process of Zn on conventional urea prills. Zn boosted wheat yield, which was greatly increased in all the Zn-amended samples, in comparison with uncoated and control treatments, as shown in **Table 3**. Grain and biological yields were maximum in the case of UZnNPs2 treatments, i.e., 4,515 ± 233 kg ha^–1^and 11,660 ± 593 kg ha^–1^, respectively. The nano-Zn amendments significantly increased the grain yields, i.e., through the addition of nano-Zn (0.5% and 4%) and molasses. The chlorophyll content, root biomass, and biological yields of all coated prills were particularly higher than the uncoated and control treatments. However, the effect was more significant for UZnNPs2 prills. The lowest increase in root biomass and biological yield was observed for UZnNPs1-treated pots. However, higher coating percentages of elemental Zn increased wheat yield and physiological attributes, including the number of grains per panicle and chlorophyll content. The control and Zn-loaded urea are depicted in **Figure 8**.

**Table 3:** Effect of different treatments on wheat plant parameters during the pot experiments.

Parameters	Units	C	UC	UZnNPs1	UZnNPs2	UZnNPs3	UZnS1	UZnS2	UZnS3
**Chlorophyll content**	SPAD	33.7 ± 1.2b	36.6 ± 1.8ab	39.7 ± 3.9ab	43.8 ± 2.3a	41.8 ± 3.4ab	40.9 ± 3.5ab	41.6 ± 1.9ab	42.9 ± 5.0ab
**Grains**	No. panicle^−1^	23.0 ± 1.2**	23.4 ± 1.9	25.0 ± 1.0	27.8 ± 1.6	25.9 ± 4.6	25.0 ± 1.0	25.3 ± 0.2	27.3 ± 1.3
**Spikelets**	No. panicle^−1^	14.9 ± 1.7**	16.6 ± 1.2	17.0 ± 0.9	18.3 ± 0.6	17.8 ± 0.9	17.1 ± 2.1	17.5 ± 0.6	17.9 ± 1.2
**100-grain weight**	g	4.3 ± 0.1b	4.5 ± 0.3ab	4.7 ± 0.2ab	5.1 ± 0.3a	4.9 ± 0.3ab	4.7 ± 0.3ab	4.8 ± 0.2ab	5.0 ± 0.4ab
**Grain yield**	kg ha^−1^	2349 ± 170d	3345 ± 375c	3459 ± 315bc	4515 ± 233a	4201 ± 187ab	3704 ± 211a-c	3849 ± 320a-c	4239 ± 319ab
**Root biomass**	kg ha^−1^	327.0 ± 27.4b	345.9 ± 22.7b	383.6 ± 63.8b	591.2 ± 70.0a	446.5 ± 53.7ab	396.2 ± 32.7b	408.8 ± 74.1b	465.4 ± 44.0ab
**Biological yield**	kg ha^−1^	6122 ± 179d	8867 ± 515c	9132 ± 502bc	11,660 ± 593a	10,993 ± 478a	9345 ± 692bc	10,566 ± 536ab	11,358 ± 549a
**Harvest index**	%	38.3 ± 1.6**	38.1 ± 5.1	37.7 ± 1.5	39.1 ± 3.9	38.5 ± 3.1	39.8 ± 1.0	36.9 ± 4.6	37.5 ± 3.2

**Non-significant.

Different letter(s) illustrate significant differences between the various treatments..

## 4 Discussion

The nanoparticles prepared from ZnSO_4_ in the present study have a similar configuration in terms of shape and size as found in previous studies (Nair et al., 2009; Larsson et al., 2019; PP, 2020; Tymoszuk and Wojnarowicz, 2020; Mahmood et al., 2021; Patella et al., 2022). The nanoparticles seen the in scanning electron micrographs were well separated, with negligible agglomeration, which is similar to the results of Umar et al. (Umar et al., 2021). The ZnO-NPs XRD spectrum presented shows clear, defined, and intense peaks, which are similar to the results of the published work of Heller et al. JCPDS, Anžlovar et al., and Umar et al. (Heller et al., 1950; JCPDS, 1977; Anžlovar et al., 2012; Umar et al., 2021). The peaks at 2Ѳ = 31.9, 34.45, 36.35, 47.6, 56.65, 62.9, 66.4, 67.9, and 69.1 are linked with (100), (002), (101), (102), (110), (103), (112) crystal planes of the hexagonal wurtzite crystal. The intense peak obtained at (002) represents the growth of wurtzite hexagonal sphere-like structures. It was previously reported that chemical synthesis and calcination steps promote the formation of hexagonal ZnO crystals (Li et al., 1999; Maret, 2009). Two bands of higher stretching vibration detected in the current study, at 3,343 and 1,435 cm^–1,^correspond to the hydroxyl (OH) groups present in ZnO-NPs. Similar peaks have been observed in the literature, and are thought to be due to the presence of hydroxyl groups (Anžlovar et al., 2012; Nagaraju et al., 2017). Metallic nanoparticles mostly yield vibrational peaks in the fingerprint region, which lies below 1,000 cm^–1^, and are due to strong interatomic vibrations. The characteristic absorption peaks, due to strong Zn–O bonds, were seen in the present study at 1,100 and 565 cm^–1^, which is in line with the previous reported studies. Moreover, it also confirms the presence of ZnO (PP, 2020).

The results of crushing strength are helpful to predict the impact resistance of prepared formulations. High values of crushing strength are favorable because of the delicate nature of prills, which can survive from the production phases until its marketing and field application (Beig et al., 2020b). The crushing strength of coated urea was highly dependent on the nature of the coating material and the percentage of the coating used. Higher coating percentages offered high values, which are the result of the improvement of nutrient delay (Babadi et al., 2015; Beig et al., 2020b; Eghbali Babadi et al., 2021). The blending of molasses with ZnSO_4_ or ZnO-NPs creates a uniform layer because of its adhesive nature (Irfan et al., 2018; Arfiana et al., 2019; Pamungkas et al., 2020). The ZnO-NPs treatments resulted in higher values of crushing strength as a result of the high surface area provided by nanoparticles. In addition, the small size and highly reactive nature of nanoparticles enhance the binding of the coating material to the surface of urea, which in turn results in better impact resistance (Kottegoda et al., 2017; Guo et al., 2018; Maghsoodi et al., 2020).

All the coating materials act as slow-release agents if applied to the outer surface of the conventional fertilizers. Coatings are thought to slow nutrient discharge whenever the urea product is immersed in water, or if it comes in contact with soil moisture. The degree of slowing of the nutrient (urea N) discharge is totally dependent on the nature of the coating material and the mechanism by which it works (Beig et al., 2020b). The coated and uncoated treatment follows either the burst release or diffusion mechanism of nutrient discharge. The initial starting phase of release follows a lag period in which a smaller quantity of nutrients is released from the urea. The second stage follows a constant discharge of nutrients, which is finally converted into a mature stage. with a gradual reduction. In the case of uncoated prills, the nutrient N is released in a catastrophic manner and follows burst nutrient discharge, i.e., the real behavior of prills. This burst discharge is due to the higher solubility of urea in the absence of any coating material. The three-stage pattern is not observed in the case of uncoated fertilizer (Beig et al., 2020b; Zafar et al., 2021). Furthermore, the Zn coatings slow the release pattern of urea N, as shown in **Figure 2A**. The water molecules first penetrate the coating layer and then the Zn coating starts to diffuse into water. The water molecules generate osmotic differences internally inside the coating layer (Zafar et al., 2021). This process can be seen in **Figure 2A**, which is similar to the release patterns demonstrated previously (Beig et al., 2020b; Beig et al., 2020c; Eghbali Babadi et al., 2021). In the current study, ZnO-NPs provided better results than ZnSO_4_ bulk salt coatings in terms of slowing the release rate. The reason for this is the higher solubility of ZnSO_4_, which, when it comes into contact with water, quickly starts to dissolve (Amrani et al., 1999; Shivay et al., 2008; Kamali et al., 2010). Conversely, the small particle size, high surface area with alleviated charge density of nanoparticles binds the nanoparticles onto the surface of urea, which then gradually releases in water. The ZnSO_4_-coated treatments showed an abrupt change in urea concentration after 12 min. From 9 to 30 min, the majority of the coating was ruptured, which released urea N following the constant release period. After this, a gradual decrease in concentration was observed. The constant 80 ppm concentration was observed during the final stage(s), which represented the complete release of urea N from all the treatments (Zafar et al., 2021). The efficiency was evaluated at 15 min for all the coated formulations using Eq. 2. The highest efficiency was demonstrated by UZnNPs2, followed by UZnS3 and UZnNPs3, i.e., 39%, 32%, and 31%, respectively. With an increasing (ZnO-NPs or ZnSO_4_) percentage, the efficiency of zinc-treated urea increases, which closely matches the findings of research conducted by Beig et al. (Beig et al., 2020c). Secondary factors that affect the efficiency of coated urea include particle size, nature, and solubility of the coating materials (Ibrahim et al., 2014; Beig et al., 2020a).

The micronutrient Zn release study is linked with release profiles of the formulated zinc-treated urea in the water–soil system. After incorporation of fertilizer treatments in the sand bed, the column was filled with deionized water. The leachate samples were collected on a daily basis for 3 consecutive days. The results of this experiment were useful to mimic and forecast the micronutrient release in the actual soil environment at low moisture contents (Tarafder et al., 2020). The highest value of Zn concentration in the leachate was observed for UZnS2 and UZnS1 treatments after 24 h, i.e., 5.39 ppm and 4.69 ppm, respectively. The release patterns of all the treatments revealed that all the Zn, coated on prilled urea, was released within 72 h from the start of the experiment. Zn release initially increased with time, but then slowed on the second day, as shown in **Figure 3A**. This increase in Zn concentration after 24 h of the experiment seems quite normal because of the presence of a porous coating layer of Zn and molasses over prills. The micronutrient Zn is released by diffusion when the coating layer comes in contact with water. This trend of nutrient release is very similar to that reported in previous published work (Yuvaraj and Subramanian, 2015; Yuvaraj and Subramanian, 2018; Tarafder et al., 2020). In contrast, the Zn release profiles of all treatments show a decreasing trend of Zn concentration after 48 h. The Zn concentration from the coated prilled urea followed a three-stage release (Yuvaraj and Subramanian, 2015; Yuvaraj and Subramanian, 2018). The concentration of Zn from all the treatments increased in samples collected after 24 h, representing the first stage of nutrient release. The second stage is represented by a sudden decrease in Zn concentration after 48 h of experimentation. In the final stage, the concentration of Zn again increased as a result of breakage of the coating film and contact of the urea core with moisture (Subramanian et al., 2008; Yuvaraj and Subramanian, 2015; Yuvaraj and Subramanian, 2018). In fact, molasses not only enhanced the micronutrient retention over prills, but also incorporated slow release into the prills (Arfiana et al., 2019; Beig et al., 2020c; Beig et al., 2020b; Pamungkas et al., 2020). This study is thus helpful to predict the nutrient release pattern that can then meet the sequential needs of crops with minimal environmental losses. In addition, the slow release of the urea N to crops enhances the long-term availability of N within the soil environment, which would boost the crop yield with fewer applications (Tarafder et al., 2020). In earlier studies, the micronutrient Zn release from zin-treated urea continued for days in agricultural fields that possessed very low moisture contents (Dou and Alva, 1998; Ge et al., 2002). The Zn and N release profiles observed in this study could, therefore, be used in the enhancement of slow-release features of existing fertilizer products.

The increased value of TOC and DOC in the present case boosts the microbial biomass content, which is similar to that observed in previous findings (Dornbush, 2007). The Zn and molasses amendment in soil with urea increased the CO_2_-C release, which then alleviated the metabolic rate (observed in the 40- and 86-daysoil samples), as demonstrated in **Figure 5A**. The addition of Zn to fields probably increased the activity of enzymes (such as dehydrogenase, cellobiohydrolase, xylosidase, and glucosidase), which gradually improved the CO_2_-C discharge rate (Sri Sindhura et al., 2014; Jośko et al., 2014; Asadishad et al., 2018). The higher discharge associated with Zn (ZnSO_4_ or ZnO-NPs) addition also increased the microbial biomass, which is similar to reported results (Khan and Scullion, 2002; Mahmood et al., 2021). The additive advantage of molasses was observed with increased values of soil TOC and DOC. The presence of molasses in the coating formulation boosts microorganism activity, which then transforms the soil carbon. This carbon fixation within the soil environment is directly linked to the soil TOC and DOC; this closely matches with the literature findings (Dębska et al., 2016). The addition of Zn and molasses to conventional urea enhanced the mineral N content of soil. The nano-Zn with molasses alleviated the mineral NO_3_^–^N content more, as seen in the present case, which is similar to the reported study earlier (Mahmood et al., 2021). Addition of nanoparticles with molasses and urea also helped to enhance the mobility of nutrients within soil that are associated with growth of microbes and enzymes (i.e., urease and phosphatase) (Raliya and Tarafdar, 2013; Raliya et al., 2016; Aziz et al., 2019). The enzymes present in the soil adjust the N available to plants (Olander and Vitousek, 2000). (Raliya and Tarafdar, 2013) Raliya and Tarafdar suggested that soil amendment of ZnO-NPs enhanced the microbe-mediated alkaline phosphatase activity, relative to the control and ZnO treatments. The same trend in Nmin values was reported in the current study, which is supported by the findings of the aforementioned research (Raliya and Tarafdar, 2013). The Zn amendment (nano or bulk) at different rates in the soil increased the Zn content of soil. Higher application rates, i.e., 4% elemental Zn (nano or bulk), resulted in higher values, which are similar to the reported results of Aziz et al. and Wang et al. (Wang et al., 2013; Aziz et al., 2019). In the present study, the addition of Zn (nano or bulk) clearly increased MBC and MBN values, similar to the findings of previous studies (Raliya and Tarafdar, 2013; Aziz et al., 2019). A few research studies summarized the application of ZnSO_4_ which could potentially be used to control urea N conversion into nitrate, which in turn is linked to urease enzyme activity (Raliya and Tarafdar, 2013).

The presence of molasses along with the Zn over prills boosted the MBC, MBN, and MBZn, which shows close correspondence with the literature (Zhang et al., 2017). The values of MBN and MBZn were increased with soil amendment of ZnO-NPs, which is similar to published studies (Aziz et al., 2019; Dimkpa et al., 2020b). The addition of Zn (nano or bulk) was interlinked with the increase in microbial growth that enhanced the mobility of essential plant nutrients for better crop growth. This can be seen by the increased plant N uptake, as shown in **Figure 7A**. The N uptake in wheat tissue was more prominent with the addition of nano-Zn than with bulk ZnSO_4_. This is because of the smaller size of the nanoparticles, which were more readily assimilated by crop roots than bulky salt particles. This in turn improves the activities of various enzymes within the plant system, especially in the case of the nanoparticle treatment, which is most likely a major cause of enhanced N uptake (Shang et al., 2019; Srivastav et al., 2021). Zn uptake in wheat tissues was significantly improved by the addition of zinc-treated urea, which is consistent with previously published studies (Aziz et al., 2019; Dimkpa et al., 2020a). The smaller particle size of nano-Zn correlates with a high level of Zn uptake in comparison with a similar amount of ZnSO_4_. The high surface area and reactivity of nanoparticles improve the absorption, dissolution, and bioavailability of Zn, which probably enhanced the Zn uptake in crops (Liu et al., 2016; Subbaiah et al., 2016; Moghaddasi et al., 2017).

The improved physiological and yield attributes clearly reflect the effect of Zn-loaded urea, which is in line with previous studies (Shivay et al., 2008; Liu et al., 2019). The main finding of the present study is that the application of ZnO-NPs could increase the number and quality of grains more than similar or higher rates of bulk ZnSO_4_ salts. This result closely matches published studies (Subbaiah et al., 2016; Dimkpa et al., 2020a). The improvement in grain yield could be linked to the higher Zn availability from the nanoparticles treatment(s), as compared with the control, uncoated, and Zn bulk salt treatments This nanoparticle application depicts its potential in reducing the fertilizer input rate without affecting the productivity and quality of plants (Umar et al., 2021; Beig et al., 2022). (Subbaiah et al., 2016) Subbaiah et al. (2016) achieved the same outcomes with ZnO-NPs as with bulk ZnSO_4_ but with fewer applications, which supports our research. The results of the present study suggest that Zn coatings also enhance the fertilizer efficiency in terms of its dissolution and, therefore, its availability in the soil (Milani et al., 2012).

## 5 Conclusions

Our study encompasses soil application of zinc-treated urea using ZnO-NPs, ZnSO_4_, and molasses for the synthesis of slow-release urea fertilizer. The fertilizer treatment of 0.5% ZnO-NPs resulted in the maximum efficiency in terms of nutrient release (N and Zn), grain yield, root biomass, and biological yield, as compared with the uncoated prills. The treatments coated with 4% bulk ZnSO_4_ also demonstrated almost equivalent values relative to the 0.5% ZnO-NPs-treated pots. A broader and valuable outcome of the present work is that a lower dose of Zn from ZnO-NPs seems superior in enhancing the crop yield and quality, relative to higher Zn input (dose) introduced from the ZnSO_4_ bulk salt. This investigation demonstrates the roles of nanotechnology in agriculture, one of which is to minimize the input of chemicals into the environment while sustaining crop yield. Nanoscale materials are expensive because of their costly manufacturing, which adds an additional expense to existing products. However, at the same time, the application of nanoparticles at lower rates is a potential cost saver for the agricultural sector and the environment. Furthermore, the process of scaling up nanoparticle manufacturing could reduce production costs and thus the retail cost of nanofertilizers. The study strongly recommends the application of slow-release urea, coated with nanodimensional Zn, to enhance fertilizer use efficiency, sustainable release of urea N, and to reduce the overall fertilizer N input. As a way forward, Zn-loaded urea should also be tested on other crops under different textured soils and climatic conditions.

## Data availability statement

The raw data supporting the conclusions of this article will be made available by the authors, without undue reservation.

## Author contributions

BB and MN conceived of the experiments. ZJ, GS, ZI, and BB carried out the experiments and analyzed the data. MZ and AH provided support during the field trials. BB, MN, and MZ wrote the manuscript with support from all co-authors. All authors contributed to the article and approved the submitted version.
